# Glysters of Alum for Dysentery, by Dr. Hamon

**Published:** 1858-04

**Authors:** 


### GLYSTERS OF ALUM FOR DYSENTERY, BY DR. HAMON.

In two epidemics of dysentery, the author has used alum very profitably. To children under ten years of age, he gives injections, containing—owing to the difference in age—from 15 to 45 grains in solution. To grown persons, he administered from 60 to 120 grains at one injection. The patient should remain perfectly quiet on the abdomen or right side after receiving the glyster, and retain it as long as possible. The alum is astringent, irritant and disinfectant in its action. The offensive and putrid evacuations become entirely odorless after a few such injections.

When the disease is in its earliest stages, a few injections he found to be enough to effect a cure, and it was always beneficial. In one parish, in which there were thirty-five patients, all recovered; and in another of forty, only two died, and they were quite old people.—*Schmidt's Jahrbucher.*

